# Salivary Lipid Peroxidation and Total Sialic Acid Levels in Smokers and Smokeless Tobacco Users as Maraş Powder

**DOI:** 10.1155/2012/619293

**Published:** 2012-04-23

**Authors:** Naciye Kurtul, Engin Gökpınar

**Affiliations:** Department of Chemistry, Division of Biochemistry, Faculty of Science, University of Kahramanmaraş Sütçü İmam, 46100 Kahramanmaraş, Turkey

## Abstract

Maraş powder (MP), a different type of smokeless tobacco (ST) and prepared from a tobacco of species *Nicotiana rustica* Linn, is widely used in Turkey. We aimed to investigate the effects of MP on salivary total sialic acid (TSA) and malondialdehyde (MDA) levels and to compare these parameters in smokers and MP users (MPUs). The salivary TSA and MDA concentrations were significantly higher in the smokers and MPU than those of control subjects and also in MPU than that of smokers. We have also observed that as the number of cigarettes consumed and MP amount increases, TSA and MDA levels increase too. In smokers, MDA values were significantly correlated with the number of cigarettes smoked and the duration of smoking. In MPU, both MDA and TSA levels were significantly correlated with the duration of MP use and the amount of daily consumed MP. We have concluded increased salivary TSA and MDA levels associated in MPU and smokers. Results can help to evaluate harmful effects of these habits. It is important to point out that bigger change in the measured parameters has been observed for MP use. This observation may be an important indication of harmful effects of ST use as MP.

## 1. Introduction

Cigarette smoking is a serious public health hazard. Smokeless tobacco (ST) is widely used as chewing tobacco and as oral snuff in the United States, Western Europe, southern parts of the Kingdom of Saudi Arabia, southern African countries, and the Sudan in northeast Africa [1–4]. Also, in Kahramanmaraş, a city located in southern Turkey, ST is widely consumed instead of cigarette smoking, and this habit has become increasingly popular among the males, especially among children and male adolescents.

Snuff is a term used to describe a wide variety of products containing finely ground tobacco as a principal constituent and other additives. Snuff is either inhaled to the nasal cavity or dipped in the oral cavity [1]. According to preparation methods snuff is called differentially in various regions of the world. In Kahramanmaraş, a different type of ST, locally called as “Maraş powder (MP)” or “oral powder,” and also “powder,” has been used for a long time. This powder is mostly preferred while trying to quit smoking or lessen it. Tobacco used for manufacture of MP is of the species *Nicotiana rustica* Linn (NRL). The leaves of a plant known as “crazy tobacco” locally are powdered, and this powder is mixed with the ash of wood especially oak, walnut, or grapevine. First of all, sun-dried leaves of this plant are powdered and mixed with the ash in approximately 1 : 2 or 1 : 3 proportions (tobacco and oak, resp.). Then, water is sprinkled onto this mixture for humidification. A small amount of this mixture, sometimes as portion-bag-packed, (approximately 1 g a quid) is applied between the lower labial mucosa and gingival for 4-5 minutes and even as long as 1-2 h. This region of the mouth has many capillary vessels; therefore, nicotine is quickly absorbed into circulation. This procedure is repeated many times during the day [2, 3].

Snuff contains a number of carcinogens [1]. The tobacco-specific nitrosamines (TSNAs) are metabolites of nicotine and are major carcinogens in tobacco products [1, 4]. Chronic inflammation may promote the carcinogenic effect of these nitrosamines through the generation of reactive oxygen species (ROS) [4]. On the other hand, ROS and lipid peroxides have been implicated in the pathogenesis of a large number of pathological states such as diabetes mellitus, atherosclerosis, heart disease, inflammation, and cancer [5, 6]. Also, inflammation is responsible for tissue injury in pathological conditions ranging from myocardial infarction to rheumatoid arthritis [7]. It has been suggested that saliva content changes rapidly in response to systemic inflammation [8, 9]. Malondialdehyde (MDA) is an indicator of lipid peroxidation (LPO), and serum and salivary MDA levels have been reported to correlate in several diseases and pathology including inflammatory conditions [10]. In addition, according to some reports, total sialic acid level (TSA) in saliva is increased with oxidative stress (OS) due to systemic or local effects [11].

On the other hand, TSA has been used as useful marker for human cancer [12–16]. Elevated concentrations of serum TSA were suggested as a potent cardiovascular risk factor in the general population [17, 18]. Also, TSA is a marker of inflammation [19]. These and other several reports indicate that there is increasing interest to sialic acid (SA) measurement in a number of branches of medicine to diagnosing, monitoring systemic health and disease states [12–14].

SA is the common name for compounds of N-acetylated derivates of neuraminic acid, which mainly occurs as nonreducing terminal residues of carbohydrate chains of glycoproteins (GPs) or glycolipids (GLs) in biological fluids and cell membranes. SAs have a central role for the function of biological systems: stabilizing the conformation of GPs and cellular membranes, assisting in cell-cell recognition and interaction and serving as chemical messengers in tissue and body fluids, affecting the function of membrane receptor molecules by developing binding sites for ligands, enzymes, and so forth, or by blocking such; affecting the functioning, stability, and survival of GPs in blood circulation. SA is also an important component of salivary GPs including IgA and other immunological and acute phase proteins [13, 19, 20].

Saliva is a complex biological fluid composed of a wide variety of organic and inorganic constituents. Interest in saliva as a diagnostic fluid has grown exponentially in recent years. This has in part been driven by the easy and safety with which saliva can be collected as compared to blood. Salivary levels of various biochemical parameters have been measured in several diseases such as infectious diseases, autoimmune diseases, cancers, and psychiatric disorders [21, 22]. Thus, it may be useful to evaluate salivary TSA and MDA levels in tobacco users.

Although, there are several reports on blood LPO [23, 24] and TSA in smokers [3, 17, 25], few studies have been performed in ST users (STU) [3]. Also, there is no report available on salivary TSA or MDA in MP users (MPU). In addition, no previous study was encountered on salivary MDA and TSA together in smokers. Therefore, the present study has been undertaken to investigate the effect of ST use as MP on both TSA and MDA levels in saliva. We also wanted to compare these parameters between the smokers and STU.

## 2. Materials and Methods

### 2.1. Subjects

The study was performed at the Department of Chemistry-Biochemistry, University of Kahramanmaraş Sütçü İmam, Turkey. Saliva samples obtained from smokers (Group II), MPU (Group III), and healthy control subjects (Group I), with the latter having never smoked and being not exposed to any passive smoking in their environment and also nonusers of MP or ST any form. Also, in Group II and Group III, there were no subjects who were both smokers and MPU. The control subjects were selected from healthy subjects, and their clinical blood profiles were within the normal range, and the general health status was normal. Individuals who are smokers and ST users were classified into subgroups with respect to the amount of consumed cigarette or oral powder as follows:

- Group II: smokers;
- Group II-A: 1–10 cigarettes/day, Group II-B: 11–20 cigarettes/day, Group II-C: >20 cigarettes/day;
- Group III: MPU;
- Group III-A: 1–10 g MP/day, Group III-B: 11–20 g MP/day; Group III-C: >20 g MP/day.

Their demographic data is presented in Table 1. All the subjects in this study were healthy men volunteers recruited from environment and university students or their friends and acquaintances. Informed consent was obtained from all subjects, who were not suffering from any disease and were not on any medications.

### 2.2. Samples

Unstimulated saliva samples were collected from each subjects after overnight, before breakfast, and after the mouth had been rinsed with distilled water. The collection was carried out at the same time of day (between 08:00 a.m. and 10:00 a.m.), and in restful and quiet circumstances. The samples were taken either later the same day or, with few exceptions, in the next few days (range 0–21 days). Saliva samples were stored at −20°C until analysis.

### 2.3. Biochemical Analyses

Saliva TSA was measured with the Denny's colorimetric method [26]. In brief, saliva samples were first hydrolyzed in H_2_SO_4_ to release SA. The periodate solution was added and incubated. The excess periodate was reduced by adding the sodium thiosulfate solution directly into the sample solution and mixing without delay. The reaction was completed by the addition of TBA solution and heating at 100°C to achieve optimum color production. The samples were cooled to room temperature in tap water. Following the addition of acidic butanol, the tubes were capped and vigorously shaken. Complete phase separation was achieved by centrifugation at 400 ×g for 5 min. The butanol phase was carefully removed and assayed colorimetrically at 549 nm. 

LPO was assayed by measurement of MDA, an end product of fatty acid peroxidation, and reacts with thiobarbituric acid (TBA) to form a colored complex that has maximum absorbance at 532 nm. In the TBA test reaction, MDA or MDA-like substances and TBA react together for production of a pink pigment having an absorption maximum at 532 nm. The reaction was performed at pH 2-3 at 90°C for 15 min. The sample was mixed cold 10% (w/v) trichloroacetic acid to precipitate protein. The precipitate was pelleted by centrifugation and an aliquot of the supernatant was reacted with an equal volume of 0.67% (w/v) TBA in a boiling water bath for 10 min. After cooling, the absorbance was read at 532 nm [27]. The concentration of MDA was calculated by the absorbance coefficient of MDA-TBA complex 1.56 × 105 cm^-1 ^M^−1^ and expressed as nmoles of MDA per mililiter saliva. 

All chemicals in this study were of analytical grade and purchased from Sigma (Stockholm) or Merck Chemicals Co. (Germany). All solutions were prepared in deionized water. 

### 2.4. Statistical Analyses

Statistical analyses were performed with the SPSS 10.0 pocket programme for Windows (SPSS Inc., Chicago, IL, USA). The data were expressed as mean values ± standard deviation (*X* ± SD). The mean values in the groups were compared with ANOVA and Tukey's HSD tests. For correlation analysis, the Pearson correlation coefficient (*r*) was used. The level of statistical significance was defined as *P* < 0.05. 

## 3. Results

In this study, salivary TSA and MDA were measured in ST users as MP and in smokers and compared with healthy controls who were nonsmokers and nonusers of ST. The results are given in Tables 1 and 2. The salivary TSA and MDA concentrations were significantly higher in the smokers (*P* < 0.001) and MPU (*P* < 0.001) than those of control subjects. Also, salivary TSA and MDA levels in MPU group were higher than those in smokers (*P* < 0.001). 

The mean salivary TSA and MDA levels were found to be lowest in the control group and highest in the MPU. The TSA and MDA levels increased with the number of cigarettes consumed and also the amount of MP used. There were no statistically significant differences in TSA levels between subgroups in smokers (*P* > 0.05) whereas significant differences were found in all subgroups of MPU (*P* < 0.05), except for the difference between Group III-A (1–10 g MP/day) and Group III-B (1–20 g MP/day) (*P* > 0.05). There were statistically significant differences in the MDA levels of all subgroups in MPU and also in smokers except for the difference between Group II-B (11–20 cigarettes/day) and Group II-C (>20 cigarettes/day). 

When compared the salivary TSA and MDA levels between subgroups of smokers and MPU, statistically significant differences were found in all groups (*P* < 0.05) except for the differences between Group III-A and Group II-B, and also Group II-C (*P* > 0.05). There was no statistically significant difference in the MDA levels between Group II-C and Group III-B (*P* > 0.05). 

In correlation analysis, there were positive and important correlations between the MDA and TSA levels of all three groups. We have also observed that as the number of cigarettes consumed and MP amount increased, the salivary TSA and MDA levels also increased. In Group II, significant correlations were observed between MDA levels and number of cigarettes smoked and duration of smoking. In Group III, both MDA and TSA levels were significantly correlated with the duration of MP use, and also correlated with the amount of daily consumed MP. 

## 4. Discussion

Our study shows that salivary TSA and MDA levels were significantly increased in smokers and MPU. This is the first study specifically meant to evaluate the salivary TSA and MDA levels together in both smokers and STU or MPU. 

ST is an alternative way of smoking. The public believes that this smokeless powder taken orally is less harmful than cigarette smoking. Also, some individuals prefer it to reduce or quit smoking. Banning smoking in public buildings, restaurants, conveyances, and many offices and workplaces also contributed to ST use as an alternative tobacco habit. 

Smoking has currently been established to be a cardiovascular risk factor [28, 29]. On the other hand, raised serum TSA concentration has been proposed to be a strong predictor of cardiovascular mortality [17, 18]. Tobacco smoke contains approximately 4000 constituents. Nicotine is one of the most pharmacologically active tobacco components with a wide range of cardiovascular effects [30]. Some investigators reported that nicotine is one of the risk factors in the development of atherosclerosis [31]. Therefore, elevated salivary TSA levels might be reflective of cardiovascular disease (CVD) risk in MPU and smokers. On the other hand, nicotine content of NRL is high. For example, nicotine content of NRL is about 6–10 fold higher than *Nicotiana tobacum* L., which is present in the cigarette tobacco [32]. In this study, salivary TSA levels in MPU were higher than those of smokers. When MP is considered to have a high nicotine content, it is obvious that harmful effects should be more pronounced. Although the reason for the association of TSA with CVD is unclear, it can be explained that a major quantity of TSA in saliva is derived from the terminal oligosaccharide chain of several of the acute-phase proteins (such as alpha 1-acid glycoprotein, alpha 1-antitrypsin, alpha 1-antichymotrypsin and ceruloplasmin which are all sialylated GPs [13, 19, 20]). It has been suggested that elevated serum TSA may reflect an acute phase response [13, 19]. An increased concentration of acute phase reactants is caused by an acute inflammatory disease or by an injury [13, 18]. Lindberg et al. showed that the positive correlation between alpha 1-antitrypsin and smoking and haptoglobin but not orosomucoid [33]. But, we did not measure the acute phase reactants, in this study. However, elevated salivary TSA levels might be reflected to CVD risk in smokers and MPU. There have been a lot of studies on serum TSA in smokers, that, generally, elevated serum TSA has been reported [3, 17, 25, 33]. However, previous reports concerning serum TSA levels in smokers are somewhat controversial. Patel et al. reported that TSA levels were not affected by smoking habit [34]. On the other hand, limited studies have been performed on serum TSA in STU and salivary TSA in smokers. Recently, we have reported that TSA levels are elevated in serum of STU as MP [3] and of smokers [25] compared to controls. Also, it has been observed that salivary TSA levels are elevated of smokers, but not significant. In addition, we have found that serum and salivary TSA levels parallel one another in alcoholics [25]. To our knowledge, there is no report available on salivary TSA in STU, so this is the first study which evaluates the salivary TSA levels in STU or MPU. 

Tobacco use has been estimated to account for 30% of the worldwide cancer burden [35]. Snuff contains a number of carcinogens, principally the most abundant ones, the TSNAs, which have been shown to be potent carcinogens in experimental animals. In addition, snuff contains other carcinogens including aliphatic and aromatic hydrocarbons, formaldehyde, ketones, alcohols, phenols, amines, amides, metals, radioelements (e.g., polonium-210, uranium-235 and 238) and polyaromatic hydrocarbons [1, 36, 37]. Several epidemiological and laboratory studies have documented that use of snuff is associated with an increased risk for cancers of the oral cavity, larynx, and pharynx [1, 36]. There are also indications for an increased risk of cancer of esophagus, pancreas, renal pelvis, and urinary bladder among snuff users [38]. In addition, smoking was reported to be a risk factor for oral cavity and esophageal cancers and liver cirrhosis as well as lung cancer [39]. The evidence so far accumulated demonstrates that tobacco habits increase endogenous N-nitroso compounds formation, thus adding to the burden of exposure by preformed carcinogenic these in tobacco products [40]. 

On the other hand, serum TSA has also been used as a tumor marker for a number of different cancers including colorectal, prostate, and breast cancers [14, 15]. Cell surfaces and membrane components play a prominent role in neoplastic behavior. Neoplasms often have an increased concentration of TSA on the tumor cell surface, and sialoglycoproteins are shed or secreted by some of these cells, which increases the concentration in blood or saliva [14]. Moreover, cancer cells have been associated with an increased activity of sialytransferase, leading to an increased amount of TSA on the cell surface, thus increasing the plasma or salivary concentration [19, 41]. TSA concentrations have been reported to be related not only to diagnosis, but also to staging, prognosis, and detection of early recurrence [14]. It has been suggested that evaluations of the serum glycoconjugate levels may be useful in early detection and staging of oral precancerous conditions and oral cancer [16] which are often associated with ST use or smoking. 

Recently, Latha et al. showed that prolonged exposure of rats to cigarette smoke resulted in significant alteration in the metabolism of GPs and glycosaminoglycans (GAGs) in different tissue [39]. SA is an important component of salivary GPs which play an important role in the properties and functions of saliva [21]. Some of these GPs are known to act as scavenger molecules [42, 43], and sialoglycoconjugates which are synthesized in salivary glands may play a role in •OH scavenging [43, 44]. Morever, it was clearly shown that the SA in the GPs is an essential moiety to scavenge •OH [43]. 

In this study, our results show that cigarette smoke or MP use caused an increase in the salivary LPO. We found that salivary MDA levels were significantly higher in tobacco groups than healthy controls, and also significantly higher in MPU than smokers. Guentsch et al. reported that smoking enhanced LPO in the saliva [45]. Also, LPO products and tobacco-derived carcinogens have been found in the saliva of tobacco chewers (35). On the other hand, saliva is reported to be suitable to detect the body's OS level [46]. 

Nicotine and other compounds in the tobacco that induce intracellular OS recognized as the important agents involved in the damage of biological molecules. These compounds are inducing many cellular processes mediated through ROS [47–49]. Some studies shown that nicotine increases ROS in a time and concentration-dependent manner. Barr and co workers have reported that as low as 0.1 *μ*M concentration of nicotine induces ROS by approximately 35%; however, significant amount of increase in ROS is observed at 1 and 10 *μ*M with 54% and 80% respectively [47]. Bagchi et al. reported that ST extract produces oxidative tissue damage [48]. Yildiz et al. demonstrated that nicotine and ST extract increased MDA generation [49]. They have assessed the generation of ROS, following treatment with 4, 0.8, and 0.08 mg of nicotine and ST extract containing the same amounts of nicotine. They have shown that all preparations of ST extract significantly increased MDA generation while only 4 mg of nicotine were sufficient to increase MDA generation. Finally, they have reported that nicotine is less toxic than ST extract that contained the same amount of nicotine. In another study, increased plasma MDA levels were reported in MPU compared with the controls [50]. Our observations confirm the results of these studies which suggest that nicotine and other components of ST increase the LPO. In our study, MDA levels were significantly correlated with the duration of MP use and the amount of daily consumed MP. At the same time, we have observed significant correlations between MDA levels and number of cigarette smoked and duration of smoking. However, Nielsen et al. found that there was significant correlation between plasma MDA and the number of hours of exposure to cigarette smoke, but no correlation between plasma MDA and the number of cigarette smoked [51]. 

In this study, salivary TSA levels paralleled with MDA levels. In MPU, both MDA and TSA levels were significantly correlated with the duration of MP use and the amount of daily consumed MP. But, in smokers, only MDA values were significantly correlated with the number of cigarettes smoked and the duration of smoking. In addition, we found a significant correlation between TSA and MDA of all three groups. On the other hand, salivary TSA level has been reported to increase with OS due to systemic or local effects [11] and plasma TSA to positively correlate with LPO [52]. ROS and other reactive species are important coordinators of the inflammatory response [5]. Cigarette smoke increases production of oxygen-free radicals by polymorphonuclear leukocytes [51]. In the literature, leukocyte counts were reported to be significantly higher in the smokers [53, 54]. Also, it has been suggested that the increased leukocyte counts, found in MPU, may be an indicator of inflammatory events in various tissues [50]. On the other hand, it has been reported that salivary MDA levels reflect circulating MDA levels well in systemic inflammatory diseases [10]. One of the possible reasons in the increase of salivary MDA may be a result of oxidative damage of the salivary glands. Possibly, continuous local irritation by tobacco can lead to injury-related chronic inflammation and OS. Morever, tissue injury can itself cause more oxidant generation which may contribute to a worsening of the injury [6]. In addition, an increase in salivary OS may be related to the alteration of salivary secretion and qualitative changes in salivary proteins. However, we did not measure these parameters, in this study. Saliva is not only the first biological fluid to encounter inhaled cigarette smoke or dipped MP, but also an important organism fluid which has closer interaction with the gastrointestinal tract. Therefore, MP has hazardous effects on the gastrointestinal tract as well as oral mucosa. Also, salivary ROS, which has been originated in both the used tobacco products and mucosal inflammation, cause oxidative damage in various cells, organs, systemic circulation, and all body. Consistent with our opinion, it has been suggested that MP could have carcinogenic effects on the oral mucosa and gastrointestinal tract, atherogenic effects on endothelial cells, and it could cause many other systemic disorders by the reason of MP increases OS [50]. 

Taking into consideration that the studies mentioned above, increased salivary TSA and MDA levels in smokers and MPU, might be related to various diseases, for example, various cancers and CVD, and both of which are also often associated with elevated TSA or MDA levels and with smoking or ST use. Morever, our findings suggest that there may be a closer interaction between the inflammatory events and smoking or MP use. It has been reported that the changes in saliva components were net effects caused by the cancer, systemic diseases, or medications as well as mucosal inflammations [55], which confirms the our opinion. 

## 5. Conclusions

From the results obtained we can conclude that increased salivary TSA associated with LPO in MPU and smokers. This may be an indication that MP use has harmful effects at least of cigarette smoking. We can say that MP may be more harmful than smoking because we found that salivary TSA and MDA levels in MPU group were higher than those in smokers. In addition to that, the correlations between the measured parameters were bigger in MPU than those in smokers. 

Besides an inflammation marker, TSA may be concluded as an alternative OS marker in tobacco exposure since the increase in TSA levels in saliva has been found to be in well accordance with MDA. Another important point is that the chronic diseases which are resulted as the harmful effects of MP usually happen later in life. Therefore, a saliva-based test could prove very useful for early detecting and monitoring of health effects of these habits since saliva is a readily available specimen. Thus, we suggest that salivary TSA and MDA may be used as useful markers for this purpose. In addition, this study is important because the finding draws attention to a significant potential public health hazard. However, we think the results of the study should be investigated further in larger samples. 

## Figures and Tables

**Table 1:** Demographic variables for the subjects and the salivary TSA and MDA levels*.

Groups	*N*	Age (years)	Duration of smoking or MP Use (years)	Consumption of cigarette (cigarettes/day) or MP (g/day)	TSA (*μ*g/mL)	MDA (nmol/mL)
Group I	30	22.23 ± 2.45	—	—	51.60 ± 3.51	0.69 ± 0.15
Group II	33	23.80 ± 3.43	6.03 ± 2.98	16.86 ± 6.78	62.60 ± 3.91^a^	1.59 ± 0.28^a^
Group II-A	10	23.60 ± 2.63	4.60 ± 2.41	9.20 ± 1.87	61.29 ± 4.32^a^	1.34 ± 0.22^a^
Group II-B	13	23.00 ± 3.53	5.85 ± 2.76	17.84 ± 2.19	62.87 ± 3.28^a^	1.65 ± 0.18^a,d^
Group II-C	10	25.57 ± 4.07	8.43 ± 2.99	26.00 ± 3.16	64.25 ± 4.33^a^	1.86 ± 0.25^a,c^
Group III	37	29.03 ± 6.46	6.03 ± 2.89	19.04 ± 7.95	75.52 ± 6.86^a,b^	2.12 ± 0.32^a,b^
Group III-A	10	29.50 ± 5.64	4.67 ± 0.81	7.50 ± 0.94	70.28 ± 2.63^a,d^	1.67 ± 0.10^a,e^
Group III-B	12	29.25 ± 7.03	4.50 ± 2.15	15.62 ± 1.49	73.04 ± 5.31^a,c,f,g^	2.02 ± 0.15^a,c,f,i^
Group III-C	15	28.67 ± 6.48	7.80 ± 3.02	26.40 ± 4.19	79.60 ± 6.93^a,c,f,g,i,k^	2.37 ± 0.21^a,c,f,g,h,j^

*Mean values ± standard deviation; *X* ± SD; TSA: total sialic acid; MDA: malondialdehyde; MP: Maraş powder.

^a^ *P* < 0.001 versus controls; ^b^ *P* < 0.001 versus Group II; ^c^ *P* < 0.001 versus Group II-AL; ^d^ *P* < 0.01 versus Group II-A; ^e^ *P* < 0.05 versus Group II-A,^f^ *P* < 0.001 versus Group II-B; ^g^ *P* < 0.001 versus Group II-C; ^h^ *P* < 0.001 versus Group III-A; ^i^ *P* < 0.01 versus Group III-A; ^j^ *P* < 0.001 versus Group III-B; ^k^ *P* < 0.01 versus Group III-B.

Group I: Controls.

Group II: Smokers.

Group II-A: 1–10 cigarettes/day; Group II-B: 11–20 cigarettes/day; Group II-C: >20 cigarettes/day.

Group III: MPU.

Group III-A: 1–10 g MP/day; Group III-B: 11–20 g MP/day; Group III-C: >20 g MP/day.

**Table 2:** Correlation coefficients.

Compared Parameters	Group I (controls)	Group II (smokers)	Group III (MPU)
Age versus TSA	0.127	−0.134	−0.022
Age versus MDA	0.124	0.205	−0.066
TSA versus MDA	0.722***	0.517**	0.756***
TSA versus consumed daily cigarette or MP		0.279	0.586***
MDA versus consumed daily cigarette or MP		0.702***	0.872***
TSA versus duration of smoking or MP use		0.268	0.564***
MDA versus Duration of Smoking or MP use		0.454*	0.474**
consumed daily cigarette or MP versus duration of smoking or MP use		0.434*	0.537***
Age versus consumed daily cigarette or MP		0.198	0.062
Age versus duration of Smoking or MP Use		0.477**	0.480**

*P < 0.05; **P < 0.01; ***P < 0.001.
